# Data-independent acquisition-based proteomics analysis correlating type 2 diabetes mellitus with osteoarthritis in total knee arthroplasty patients

**DOI:** 10.1097/MD.0000000000028738

**Published:** 2022-02-04

**Authors:** Lulu Zhao, Tong Wu, Jiayi Li, Chunyan Cai, Qingqiang Yao, Yi-Shen Zhu

**Affiliations:** a School of Pharmaceutical Sciences, Nanjing Tech University, Nanjing, Jiangsu Province, PR China; b College of Biotechnology and Pharmaceutical Engineering, Nanjing Tech University, Nanjing, Jiangsu Province, PR China; c Department of Orthopedic Surgery, Nanjing First Hospital of Nanjing Medical University, Nanjing, Jiangsu Province, PR China; d Key Lab of Additive Manufacturing Technology, Institute of Digital Medicine, Nanjing Medical University, Nanjing, Jiangsu Province, PR China.

**Keywords:** chronic inflammation, diabetes mellitus, osteoarthritis, oxidative stress, proteomics

## Abstract

**Background:**

To explore the effects of type 2 diabetes mellitus (T2DM) on osteoarthritis (OA), 12 bone tissue samples were obtained surgically from the human total knee arthroplasty patients and analyzed by quantitative proteomics.

**Methods:**

Based on patient clinical histories, patient samples were assigned to diabetes mellitus osteoarthritis (DMOA) and OA groups. A data-independent acquisition method for data collection was used with proteomic data analysis to assess intergroup proteomic differences. Gene Ontology (GO) functional analysis and Kyoto Encyclopedia of Genes and Genome pathway enrichment analysis were used to further find the correlation between T2DM and OA.

**Results:**

GO functional analysis found 153 differentially expressed proteins between DMOA and OA groups, of which 92 differentially expressed proteins were significantly up-regulated and 61 were significantly down-regulated. Kyoto Encyclopedia of Genes and Genome pathway analysis found 180 pathways, including 9 pathways significantly enriched. Further data analysis revealed that 6 signaling pathways were closely associated with T2DM and OA.

**Conclusion:**

OA and DMOA onset and progression were closely related to synthesis and metabolism of extracellular matrix components (e.g., fibronectin, decorin, etc.). The effects of T2DM on OA occur though 2 major ways of oxidative stress and low-grade chronic inflammation, involving in 2 inhibited signaling pathways and 4 activated signaling pathways.

## 1 Introduction

Osteoarthritis (OA) is a whole joint disease with early modifications of synovium and subchondral bone.^[1]^ Worldwide estimates are that 9.6% of men and 18.0% of women aged ≥60 years have symptomatic OA. For advanced OA patients who have difficulties to walk and move,^[2,3]^ total knee arthroplasty is frequently recommended to improve quality of life.

During a long time, ageing and mechanical stress were considered as the single risk factors of OA. However, recent advances in the knowledge of OA had highlighted the involvement of metabolic syndrome factors. Such factors working together appear to destroy the balance between synthesis and degradation of articular cartilage cells, extracellular matrix (ECM) components, and subchondral bone.^[4–6]^

As an important pathological component of metabolic syndrome,^[7]^ diabetes mellitus (DM) is divided into 4 types: type 1, type 2, other special types, and gestational DM. Among them, type 1 DM and type 2 DM (T2DM) are the main types, and T2DM patients account for more than 90% of diabetic patients. Epidemiological research studies have demonstrated that OA and T2DM are closely related.^[4–6]^ Data shows that the OA incidence rate in diabetic patients is 30%, a rate more than double that of non-diabetic patients (13%).^[8]^ Meanwhile, there are numerous overlaps between OA-related signaling pathways and T2DM-related signaling pathway (Table 1),^[9–13]^ indicating that OA and T2DM are not only closely related, but may engage in extensive interactions as well.

**Table 1:** Signaling pathways related to osteoarthritis and type 2 diabetes mellitus.

	Signaling pathways related to OA	Signaling pathways related to DM
1	PI3K-Akt signaling pathway	PI3K-Akt signaling pathway
2	MAPK signaling pathway	AMPK signaling pathway
3	NF-κB signaling pathway	Insulin signaling pathway
4	AMPK signaling pathway	p53 signaling pathway
5	Wnt signaling pathway	mTOR signaling pathway
6	Notch signaling pathway	NF-κB signalng pathway
7	Toll-like receptor signaling pathway	VEGF signalng pathway
8	p65 signaling pathway	Calcium signaling pathway
9	mTOR signaling pathway	MAPK signaling pathway
10	TGF-β1 signaling pathway	HIF-1 signaling pathway
11	HIF-1 signaling pathway	Ras signaling pathway
12	Jak2-STAT3 signaling pathway	Jak-STAT signaling pathway
13	Hedgehog signaling pathway	TGF-β1 signaling pathway
14	IL-17 signaling pathway	cGMP-PKG signaling pathway
15	FoxO signaling pathway	TNF signaling pathway
16	VEGF signaling pathway	cAMP signaling pathway
17	Estrogen signaling pathway	PPAR signaling pathway
18	ANP32A/ATM signaling pathway	Caspase signaling pathway
19	AGE-RAGE signaling pathway in diabetic complications	Adipocytokine signaling pathway
20	PERK/Bip signaling pathway	Toll-like receptor signaling pathway
21	SDF-1/CXCR4 signaling pathway	NOD-like receptor signaling pathway
22	Hippo-YAP signaling pathway	FoxO signaling pathway
23	OPG-RANK-RANKL signaling pathway	Sphingolipid signaling pathway
24	Integrin-actin signaling pathway	Fc epsilon RI signaling pathway
25	BMP signaling pathway	g-secretase mediated ErbB4 signaling
26	SOX9 signaling pathway	
27	IGF signaling pathway	
28	p53 signaling pathway	

^∗^DM = diabetes mellitus, OA = osteoarthritis.

Data-independent acquisition (DIA) is a new tandem mass spectrometry method that fragments and analyzes all peptide ions within a selected mass-to-charge ratio range. Because of its higher throughput,^[14]^ greater reproducibility,^[14]^ precision,^[15]^ and accuracy^[15]^ than previous MS methods, DIA analysis technology has been embraced by medical practitioners in recent years for use in clinical applications.^[16,17]^ In this study we implemented DIA-based proteomic analysis of specimens obtained surgically from 12 total knee arthroplasty patients. DIA results combined with clinical laboratory results and medical histories were then analyzed together to reveal significant differences between proteomes of diabetes mellitus osteoarthritis (DMOA) and OA patient groups and pave the way for further understanding of the effects of T2DM on OA.

## 2 Material and methods

### 2.1 Samples collection

Human bone tissue samples of all OA patients with or without T2DM whose surgery date was between May 7, 2020, and May 20, 2020, were collected at Nanjing First Hospital. Detailed patient information is summarized in Table 2. No patients received neoadjuvant-based radiological or chemotherapeutic treatments prior to undergoing surgical resection. Participants signed a written informed consent form, and this study design was approved by the Medical Ethics Committee of the Ethical Committee of Nanjing First Hospital (reference number: KY20170109–04).

**Table 2:** Clinical Information of 12 samples.

Characteristics	DMOA	OA
Subjects, N	4	8
Gender (M/F)	0/4	1/7
Age (mean ± SD)	74 ± 3	74 ± 6
BMI (mean ± SD)	26.2 ± 1.6	26.4 ± 1.8
Diagnoses	OA	OA
Type 2 diabetes mellitus	yes	no
Position of samples (right/left)	the knee joint (2/2)	the knee joint (3/5)
Degree of OA (III/IV)	2/2	5/3

^∗^BMI = body mass index, DMOA = diabetes mellitus osteoarthritis, OA = osteoarthritis; Degree of OA is graded according to the Kellgreen grading standard.

### 2.2 Protein extraction and peptide preparation for liquid chromatography-mass spectrometry

After surgical resection, 12 bone tissue samples of the same mass at the distal femur were taken and homogenized using a homogenizer (24 × 2, 6.0 m/s, 60 s, twice), followed by addition of SDT buffer (4% sodium dodecyl sulfate, 100 mM dithiothreitol, 150 mM Tris-HCl, pH 8.0) (Bio-Rad, Hercules, California, USA). After centrifugation and quantification using a BCA assay Kit (Bio-Rad, Hercules, California, USA), an equal amount of protein from each sample was taken and mixed to create a pooled sample for library generation and quality control (QC). 200 μg of protein was dissolved in SDT buffer. Detergent, dithiothreitol, and other low-molecular weight components were removed using UA buffer (8 M urea, 150 mM Tris-HCl pH 8.0) (Bio-Rad, Hercules, California, USA) followed by repeated ultrafiltration (10-kD cutoff). Next, 100 μL of 100 mM iodoracetamide (Bio-Rad, Hercules, California, USA) was added then samples were incubated. Filters were washed with UA buffer triplicates. Finally, protein suspensions were digested with 4 μg trypsin (Promega, Madison, Wisconsin, USA) overnight at 37°C. Filtrates were collected and desalted using an Empore SPE Cartridges C18 (standard density, bed I.D. 7 mm, volume 3 mL), followed by concentrating and reconstituting in 0.1% (v/v) formic acid (Sigma, St.Louis, Missouri, USA). Peptide content was estimated via UV light absorption-based spectral density determinations at 280 nm using an extinction coefficient of 1.1 of 0.1% (g/L) solution that was calculated based on the frequency of tryptophan and tyrosine residues within vertebrate proteins. Pooled peptides after digestion were then fractionated to create 10 fractions using a Thermo Scientific Pierce High pH Reversed-Phase Peptide Fractionation Kit. Each fraction was concentrated and reconstituted followed by desalting and reconstituting in 40 μL of 0.1% (v/v) formic acid. Next, iRT-Kits (Biognosys, BezirkDietikon, Kanton Zürich, Switzerland) were employed to correct for relative retention time differences between runs; a 1:3 volume ratio (of iRT standard peptides to sample peptides) was used.

### 2.3 Liquid chromatography-tandem mass spectrometry analysis of pooled sample

All fractions for library generation were injected on a Thermo Scientific Q Exactive HF X mass spectrometer connected to an Easy nLC 1200 chromatography system (Thermo Scientific). Each fraction (1.5 μg) was first loaded onto an EASY-Spray C18 trap column (Thermo Scientific, P/N 164946, 3 μm, 75 μm × 2 cm), then peptides were separated using an EASY-Spray C18 LC analytical column (Thermo Scientific, ES802, 2 μm, 75 μm × 25 cm) with a linear gradient of 84% acetonitrile (Merck, Hunterdon County, New Jersey, USA) and 0.1% formic acid at a flow rate of 250 nL/min for 90 minutes. The MS detection method: positive ion; the scan range: 300 to 1800 m/z; the resolution for MS1 scans: 60000 at 200 m/z; the target of automatic gain control: 3e6; maximum injection time (IT) 25 ms; and dynamic exclusion: 30.0 second. Each full MS-single ion monitoring (SIM) scan followed 20 ddMS2 scans. Resolution for MS2 scans: 15000; automatic gain control (AGC) target: 5e4; maximum IT25 ms; and normalized collision energy: 30 eV.

### 2.4 Liquid chromatography-mass spectrometry analysis of 12 samples

Sample peptides were analyzed in the DIA mode. Each DIA cycle contained 1 full MS-SIM scan and 30 DIA scans and the mass range was 350 to 1800 m/z using the following settings: SIM full scan resolution: 120,000 at 200 m/z; AGC 3e6; maximum IT50 ms; profile mode. DIA scan settings were: resolution of 15,000; AGC target 3e6; Max IT auto; normalized collision energy 30 eV. Running time, flow rate and mobile phase are the same as the previous part. QC samples were injected using DIA mode from the beginning of the MS study throughout completion of 6 injections during the experiment in order to monitor MS performance.

### 2.5 Database searches and spectral library construction

To construct the DDA library, the FASTA sequence database was searched using Spectronaut Pulsar X (version14.4, Biognosys AG, USA) after FASTA database download from the UniProt website (http://www.uniprot.org) with the iRT peptides sequence added (>Biognosys|iRTKit|Sequence_fusionLGGNEQVTRYILAGVENSKGTFIIDPGGVIRGTFIIDPAAVIRGAGSSEPVTGLDAKTPVISGGPYEYRVEATFGVDESNAKTPVITGAPYEYRDGLDAASYYAPVRADVTPADFSEWSKLFLQFGAQGSPFLK). The parameters were set as follows: enzyme set to trypsin, max missed cleavage set to 2, fixed modification set to carbamidomethyl (C), dynamic modification set to oxidation(M) and acetyl (Protein N-term). All protein identification results were evaluated then significant results were selected based on 99% confidence, as determined using false discovery rate (FDR =N(decoy)^∗^2/(N(decoy)+ N(target))) ≤1%. The spectral library was constructed by importing original raw spectral files and search results into Spectronaut Pulsar X (version14.4, Biognosys AG, USA). DIA data were analyzed by searching the abovementioned constructed spectral library. Main software parameters were set as follows: retention time prediction type is dynamic iRT, interference on MS2 level correction is enabled, and cross run normalization is enabled. All results were filtered based on the *P* value cutoff of .01 (equivalent to FDR <1%).

### 2.6 Data extraction and statistical analysis

**Figure 1:**
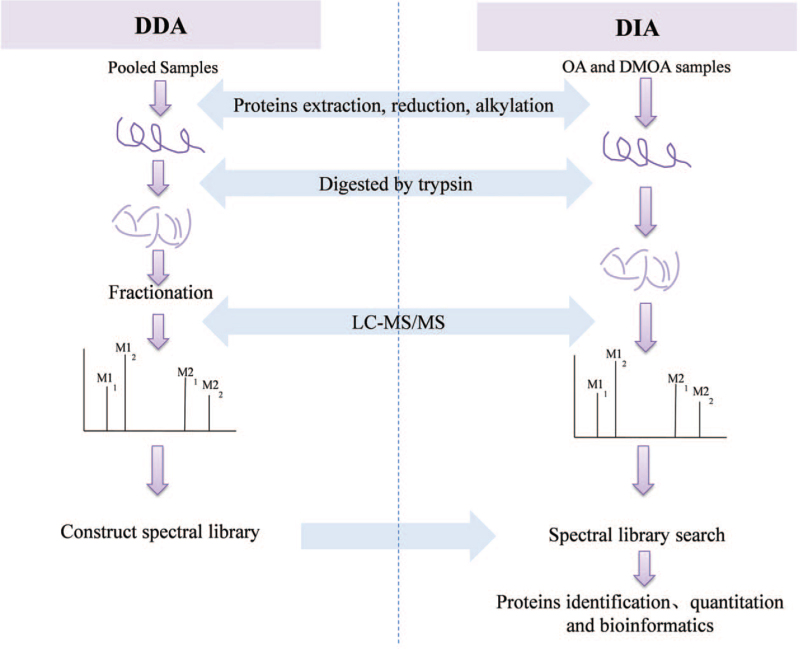
The experiment and analysis flow chart.

Cluster 3.0 (http://bonsai.hgc.jp/∼mdehoon/software/cluster/software.htm, version 3.0, Sun microsystems, USA) and Java TreeView software (http://jtreeview.sourceforge.net, version 3.0, Sun microsystems, USA) were used to perform hierarchical cluster analyses. The Euclidean distance algorithm for similarity measurements and average linkage clustering algorithm (clustering using centroids of the observations) used for cluster analysis were selected when performing hierarchical clustering analysis. A heat map was often generated for use as a visual aid in addition to dendrograms. Protein sequences of selected differentially expressed proteins (DEPs) were locally searched using NCBI BLAST+ client software (ncbi-blast-2.2.28+-win32.exe) and InterProScan to find homologous sequences, then Gene Ontology (GO) terms were mapped and sequences were annotated using the software program Blast2GO (https://www.blast2go.com/, version 5.2.5, BioBam, USA). GO annotation results were plotted using R package scripts. After completion of annotation steps, subject proteins were blasted against the online Kyoto Encyclopedia of Genes and Genome (KEGG) database (http://geneontology.org/) to retrieve their KEGG orthology database matches that were subsequently mapped to KEGG pathways. The application of enrichment analyses was based on the Fisher exact test while considering the whole quantified proteins as the background dataset. The Benjamini-Hochberg correction for multiple tests was further applied to adjust derived *P* values to ensure that only functional categories and pathways with *P* values below the threshold of .05 were considered significant. Protein-protein interaction (PPI) information for selected proteins was retrieved from the IntAct molecular interaction database (http://www.ebi.ac.uk/intact/) according to their gene symbols was obtained using STRING database (http://string-db.org/). The results were downloaded in XGMML format and imported into Cytoscape (https://cytoscape.org/, version 3.2.1, JetBrains, Czech Republic) to visualize and further analyze functional protein-protein interaction networks. As a final analysis, the node degree of each protein was calculated to evaluate the relative importance of the protein within the PPI network. The experimental and analytical flow chart was shown in Figure 1.

## 3 Results

### 3.1 MS analysis of the pooled samples and 12 clinical samples

**Figure 2:**
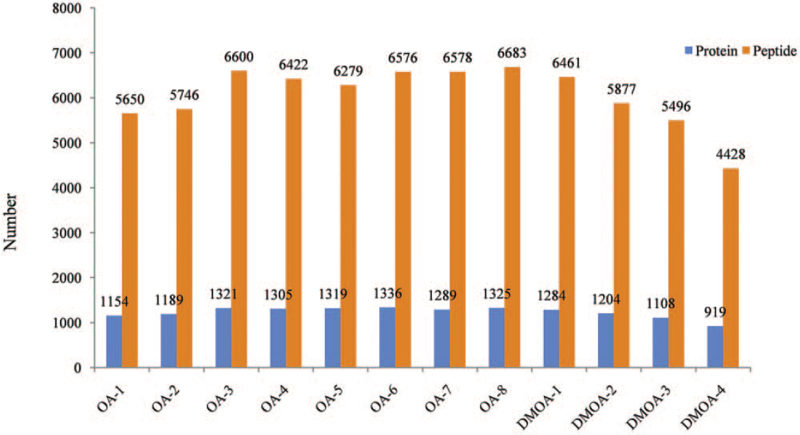
Numbers of proteins and peptides quantified in 12 clinical samples. OA 1–8 represents 8 osteoarthritis patients; DMOA 1–4 represents 4 type 2 diabetes mellitus osteoarthritis patients.

Pooled sample was analyzed as triplicates designated QC-1, QC-2, and QC-3. Numbers of peptides quantified were 6405, 6323, and 6227, respectively, yielding a relative standard deviation value of 1.15%. And numbers of parent proteins were 1269, 1281, and 1250 proteins, respectively, yielding a relative standard deviation value of 1.01% (Supplementary Digital Content Tables S1–S2). Correlation coefficients of QC-1, QC-2 and QC-3>0.9 and all QC samples were centrally distributed, yielding a mean coefficient of variation (CV) value of 13.02%. The number of peptides and proteins quantified of 12 samples was shown in Figure 2.

### 3.2 Quantification of protein expression differences between proteomes of OA and DMOA groups

**Figure 3:**
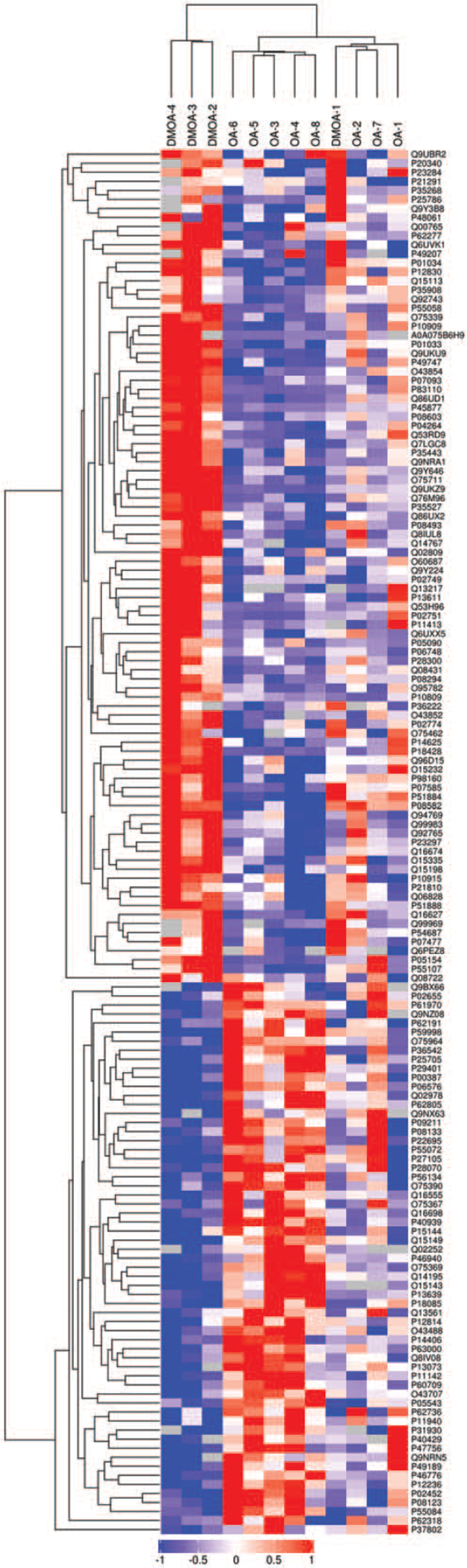
Relative expression levels of 153 differentially expressed proteins. The ordinate represents a significantly differently expressed protein; the abscissa shows patient sample designations. Values of the logarithm of expression levels of significantly differently expressed proteins in different samples (Log2 Expression) were depicted using different colors in the heat map; red represents a significantly up-regulated protein, blue represents a significantly down-regulated protein, and gray represents no protein quantitative information.

**Figure 4:**
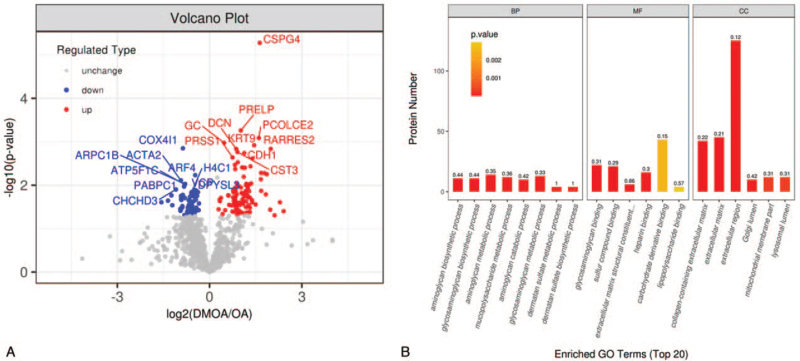
(A) Volcano map analysis of 153 differentially expressed proteins (DEPs). Blue dots represent significantly up-regulated proteins, red dots represent significantly down-regulated proteins, and gray dots represent proteins with no expression difference. (B) Gene Ontology (GO) terms enrichment analysis of 153 DEPs. The label indicates the enrichment factor which represents the ratio of the number of DEPs annotated to a GO functional category to the number of all identified proteins annotated to the GO functional category. BP = biological process, CC = cellular component, MF = molecular function.

DIA MS was used to quantitatively analyze differences in protein expression between DMOA and OA groups (fold change<0.8 or >1.2 and *P* value<.05). A total of 153 DEPs were found, of which 92 DEPs were significantly up-regulated and 61 were significantly down-regulated (Fig. 3 and Fig. 4A); in the DMOA group, levels of some DEPs associated with functional terms of signaling pathways, glycoprotein, secreted, etc. were increased, while levels of proteins associated with functional terms of acetylation, ATP synthesis, phosphoprotein, transport, etc. were decreased. Next, GO term enrichment analysis was performed (Fig. 4b, Supplementary Digital Content Table S3). Terms within the biological process (BP) GO functional category that exhibited significant intergroup differences included mucopolysaccharide metabolic process (12 DEPs), aminoglycan metabolic process (14 DEPs), glycosaminoglycan biosynthetic process (11 DEPs), aminoglycan biosynthetic process (11 DEPs), and glycosaminoglycan metabolic process (13 DEPs). Terms associated with the molecular function (MF) GO functional category that exhibited significant intergroup differences included glycosaminoglycan binding (22 DEPs), heparin binding (16 DEPs), sulfur compound binding (21 DEPs), extracellular matrix structural constituent conferring compression resistance (6 DEPs), and carbohydrate derivative binding (43 DEPs). Terms associated with the cellular component (CC) GO functional category that exhibited significant intergroup differences included ECM (45 DEPs), collagen-containing ECM (42 DEPs), Golgi lumen (10 DEPs), mitochondrial membrane part (12 DEPs), and extracellular region (125 DEPs).

### 3.3 KEGG pathway enrichment analysis of differentially expressed proteins (DEPs)

Ultimately, 153 DEPs were enriched within 180 KEGG pathways (Supplementary Digital Content Table S4). Based on *P* value <.05, a total of 9 pathways were significantly enriched, including adherens junction, proteoglycans in cancer, thermogenesis, focal adhesion, oxidative phosphorylation, regulation of actin cytoskeleton, amyotrophic lateral sclerosis, valine, leucine and isoleucine degradation, and ECM-receptor interaction. Functional analysis of these 9 pathways revealed that they were mainly associated with cell communication (33.33%), diseases (22.22%), metabolism (22.22%), organismal systems (11.11%), and cellular processes (11.11%).

### 3.4 Protein-protein interaction analysis of differentially expressed proteins

**Figure 5:**
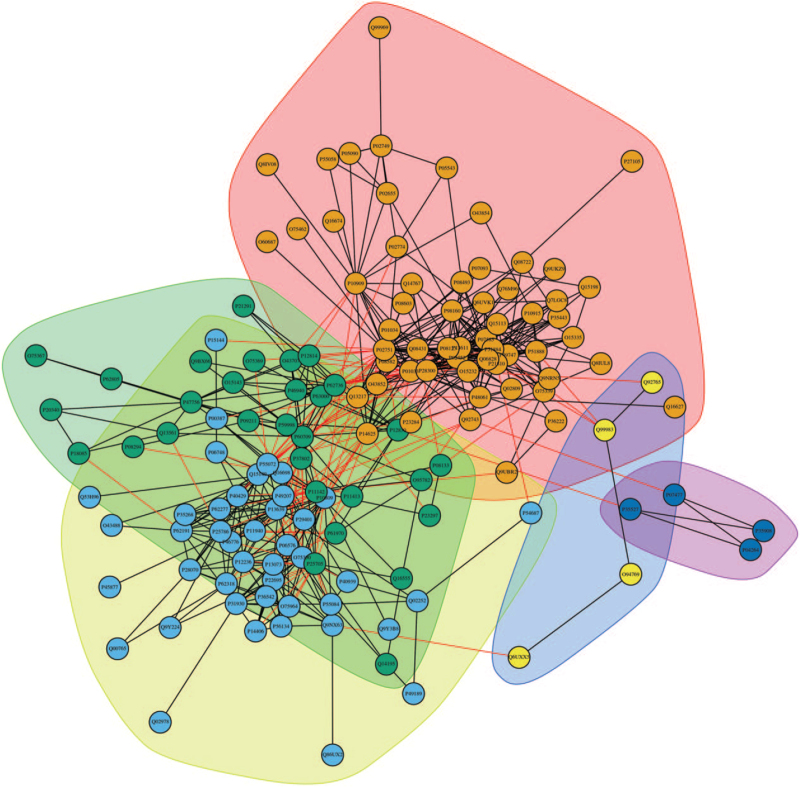
The protein-protein interaction (PPI) network of 139 differentially expressed proteins (DEPs).

The PPI network of DEPs contained 139 DEPs (14 DEPs lacked protein interactions), and 627 edges representing interactions between proteins (Fig. 5). The average node degree in the network was 8.25 and the average local clustering coefficient was 0.486. The PPI network was mainly divided into 5 functional modules. Module 1 (pink) included 58 nodes and 259 edges that were mainly related to ECM-receptor interaction, metabolism and PI3K-AKT signaling pathway. Module 2 (yellow) included 43 nodes and 161 edges that were mainly related to metabolism of amino acids and derivatives, oxidative phosphorylation, and pentose phosphate pathway. Module 3 (cyan) included 30 nodes and 76 edges that were related to insulin signaling pathway and MAPK signaling pathway. Module 4 (blue) included 4 nodes and 3 edges that were related to WNT signaling pathway and ECM. Module 5 (lavender) included 4 nodes and 5 edges that were related to protein digestion and absorption.

### 3.5 Further analysis of differentially expressed proteins related to T2DM or OA signaling pathways

**Figure 6:**
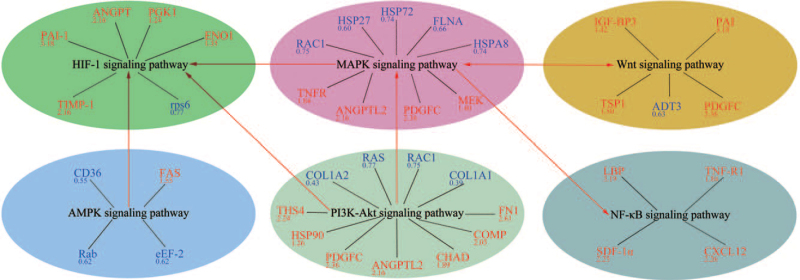
Protein-pathway interaction networks of distinct proteins. Red text indicates up-regulated proteins and blue text indicates down-regulated proteins, while numbers represent fold changes. Red lines connecting ellipses represent activation.

Further data analysis was conducted to understand functional roles of DEPs that were associated with T2DM or OA signaling pathways. Subsequently, 7 signaling pathways related to T2DM and OA were identified (Table 3, Fig. 6).

**Table 3:** Two down-regulated and four up-regulated signaling pathways in the diabetes mellitus osteoarthritis group.

	Signaling pathways	Differently expressed proteins
1	PI3K-Akt	FN1 (2.61), PDGFC (2.36), THS4 (2.24), ANGPTL2 (2.16), COMP (2.05), CHAD (1.89), HSP90 (1.26), RAS (0.77), RAC1 (0.75), COL1A2 (0.43), COL1A1 (0.39).
2	AMPK	FAS (1.55), EEF2 (0.63), RAB (0.62), CD36 (0.55).
3	MAPK	PDGFC (2.36), ANGPTL2 (2.16), TNFR (1.84), MEK (1.40), RAC1 (0.75), HSPA8 (0.74), HSP72 (0.74), FLNA (0.60), HSP27 (0.60).
4	Wnt	PAI-1 (3.18), PDGFC (2.36), TSP1 (1.50), IGF-BP3 (1.16), ADT3 (0.63).
5	HIF-1	PAI-1 (3.18), ANGPTL2 (2.16), TIMP-1 (2.16), ENO1 (1.35), PGK1 (1.24), rpS6 (0.77).
6	NF-κB signaling pathway	LBP (3.19), CXCL12 (2.26), SDF-1α (2.25), TNFR (1.84).

^∗^ANGPT = angiopoietin, ANGPTL2 = angiopoietin-like protein 2, CD36 = platelet glycoprotein 4, CHAD = chondroadherin, COL1A1 = collagen alpha-1(I) chain, COL1A2 = collagen alpha-2(I) chain, COMP = cartilage oligomeric matrix protein, CXCL12 = stromal cell-derived factor 1, EEF = eukaryotic elongation factor, ENO1 = enolase-α, FAS = Fatty acid synthase, FLNA = filamin-A, FN1 = fibronectin 1, HSP27 = heat shock protein 27, HSP72 = heat shock protein 72, HSP90B1 = endoplasmin, HSPA8 = heat shock cognate 71 kDa protein, IGF-BP3 = insulin-like growth factor-binding protein 3, LBP = lipopolysaccharide-binding protein, LDHA = lactate dehydrogenase A, MEK = mitogen-activated protein kinase kinase, PAI-1 = plasminogen activator inhibitor type 1, PDGFC = platelet-derived growth factor C, PGK1 = phosphoglycerate kinase 1, RAB = ras-related protein, RAC1 = ras-related C3 botulinum toxin substrate 1, RAS = rat sarcoma, rpS6 = 40S ribosomal protein S6, SDF-1α = stromal cell derived factor alpha-1, THBS4 = thrombospondin-4, TIMP-1 = tissue inhibitor of metalloproteinase 1, TNF-R1 = tumor necrosis factor receptor 1, TSP-1 = thrombin sensitive protein 1.

## 4 Discussion

Scanning of pooled sample triplicates using the DDA method generated average CV values that were <15%, with correlation coefficients that were all >0.9, together demonstrated that the experimental methodology has satisfactory reproducibility and stability. Peptide could be accurately integrated and quantified by collecting 5 data points at peptide chromatographic peaks. There was no significant difference in the number of proteins between the DMOA group and the OA group, indicating that the parallelism among the 12 samples was good. Moreover, Figure 3 demonstrated that comparisons of DEPs between patients within a single group revealed significantly more similarity overall than comparisons of DEPs between patients of different groups, indicating that grouping patients according to T2DM status was a sound strategy for studying T2DM effects on OA.

According to GO terms enrichment analysis results, of the first 5 biological process terms, 3 were metabolic processes of ECM and 2 were biosynthetic processes of ECM. And in the PPI network, all 5 functional modules are related to ECM or amino acid and protein metabolism. In other words, T2DM may induce imbalances of ECM synthesis and degradation that promote OA progression.^[18,19]^ Functional analysis results of significantly enriched pathways highlighted the key role that cell communication plays in development of both T2DM and OA and are consistent with results of other studies showing that both T2DM onset and development involve complex signaling between pancreatic islet cells and hormones, regulatory factors, and metabolites released from peripheral tissues (e.g., fat,^[20]^ liver,^[21–23]^ and skeletal muscle).^[24–26]^

Two inhibitory signaling pathways and 4 activation signaling pathways were discovered in further data analysis (Fig. 6). Down-regulation of both RAC1 and RAS indicated that PI3K-AKT signaling pathway was inhibited in DMOA patients.^[27,28]^ Inhibited PI3K-AKT signaling pathway can interfere with glucose homeostasis and lipid metabolism,^[29]^ leading to insulin resistance (IR),^[30]^ and both T2DM occurrence and progression.^[31]^ Meanwhile, down-regulation of FLNA and HSP72 activated MAPK signaling pathway, which marks the occurrence of oxidative stress. Oxidative stress can disrupt the function of β cells in the islets, leading to IR and eventually T2DM development.^[32]^ Moreover, up-regulation of PAI-1, TSP1, and IGF-BP3 activated p53 signaling pathway. Notably, LBP, TNF-R1, and SDF-1α were all up-regulated as evidence that NF-κB signaling pathway was activated. Activated NF-κB signaling pathway aggravates OA by promoting inflammatory response and triggering expression of proteins whose activities promoted joint destruction.^[33]^ Up-regulation ofTIMP-1, PAI-1, etc. activated HIF-1 signaling pathway, whose abnormal activation influenced chondrocyte activity, cartilage matrix synthesis,^[34]^ and induced T2DM development by promoting anaerobic metabolism and inhibiting the tricarboxylic acid cycle.^[35]^ In addition, down-regulation of RAB led to down-regulation of GLUT4, which reduced glucose uptake and increased blood sugar, indicating that AMPK signaling pathway was inhibited in T2DM. The down-regulation of AMPK expression increased FAS expression and promoted fatty acid biosynthesis.^[36]^ It is worth noting that the up-regulation or down-regulation of some proteins is the result of the joint action of multiple signaling pathways, so the expression of these proteins differing between groups might not be as indicative as proposed. In the future, we will further verify correlating between T2DM and OA by in vivo and in vitro experiments. Moreover, we will also collect and analyze more clinical samples.

In postoperative follow-up visits and patient inquiries, we found that blood sugar levels of DMOA patients can be maintained within the normal range through diet, exercise, and rational use of medications. If nondiabetic OA patients had a long-term high-sugar diet and lack of exercise, hyperglycemia may occur, which in turn stimulated DM and OA-related signaling pathways and aggravated OA. We therefore recommend that orthopedic clinicians include detailed medical histories in OA patient records and ensure that blood glucose levels of DMOA patients are monitored and documented. Such measures would alert clinical caregivers to changes in patient DM status and help caregivers formulate reasonable diagnosis and treatment plans.

## 5 Conclusions

DIA method with proteomic data analysis was used to assess intergroup proteomic differences. A total of 153 DEPs were found, and 6 signaling pathways constituted a complex system that affected each other were analyzed. The effects of T2DM on OA occur though 2 major ways of oxidative stress and low-grade chronic inflammation. Inhibiting the 2 pathways could slow OA progression and benefit OA patients. At the same time, we also provide an open data set for use in analysis of signaling pathways in future DM and OA patient specimens. Such studies will enhance our understanding of molecular mechanisms underlying DM and OA diseases and disease interactions.

## Author contributions

**Conceptualization:** Lulu Zhao, Qingqiang Yao, Yishen Zhu.

**Data curation:** Lulu Zhao, Yishen Zhu.

**Funding acquisition:** Jiayi Li, Qingqiang Yao, Yishen Zhu.

**Investigation:** Tong Wu, Chunyan Cai.

**Resources:** Jiayi Li, Qingqiang Yao.

**Visualization:** Lulu Zhao.

**Writing – original draft:** Lulu Zhao, Tong Wu, Chunyan Cai, Yishen Zhu.

**Writing – review & editing:** Lulu Zhao, Qingqiang Yao, Yishen Zhu.

## Supplementary Material

Supplemental Digital Content

## Supplementary Material

Supplemental Digital Content

## Supplementary Material

Supplemental Digital Content

## Supplementary Material

Supplemental Digital Content
